# Genetic alteration of heparan sulfate in CD11c + immune cells inhibits inflammation and facilitates pathogen clearance during influenza A virus infection

**DOI:** 10.1038/s41598-022-09197-7

**Published:** 2022-03-30

**Authors:** So Young Kim, Purva Gupta, Scott C. Johns, Elina I. Zuniga, John R. Teijaro, Mark M. Fuster

**Affiliations:** 1 Division of Pulmonary and Critical Care, Department of Medicine, University of California San Diego, La Jolla, CA USA; 2 VA San Diego Healthcare System, Medical and Research Sections, La Jolla, CA USA; 3 Division of Biological Sciences, University of California San Diego, La Jolla, CA USA; 4 The Scripps Research Institute, La Jolla, CA USA; 5 Glycobiology Research and Training Center, University of California San Diego, La Jolla, CA USA; 6 Veterans Medical Research Foundation, San Diego, CA USA; 7 UCSD Department of Medicine, Division of Pulmonary & Critical Care, VA San Diego Healthcare System, 3350 La Jolla Village Drive, San Diego, CA 92161-111J USA

**Keywords:** Glycobiology, Translational research, Infection

## Abstract

Survival from influenza A virus (IAV) infection largely depends on an intricate balance between pathogen clearance and immunomodulation in the lung. We demonstrate that genetic alteration of the glycan heparan sulfate (HS) in CD11c + cells via *Ndst1f*/f *CD11cCre* + mutation, which inhibits HS sulfation in a major antigen presenting cell population, reduces lung inflammation by A/Puerto Rico/8/1934(H1N1) influenza in mice. Mutation was also characterized by a reduction in lung infiltration by CD4^+^ regulatory T (T_reg_) cells in the late infection/effector phase, 9 days post inoculation (p.i.), without significant differences in lung CD8 + T cells, or T_reg_ cells at an earlier point (day 5) following infection. Induction of under-sulfated HS via Ndst1 silencing in a model dendritic cell line (DC2.4) resulted in up-regulated basal expression of the antiviral cytokine interferon β (IFN-β) relative to control. Stimulating cells with the TLR9 ligand CpG resulted in greater nuclear factor-κB (NFκB) phosphorylation in Ndst1 silenced DC2.4 cells. While stimulating cells with CpG also modestly increased IFN-β expression, this did not lead to significant increases in IFN-β protein production. In further IFN-β protein response studies using primary bone marrow DCs from *Ndst1f*/f *CD11cCre* + mutant and *Cre*− control mice, while trace IFN-β protein was detected in response to CpG, stimulation with the TLR7 ligand R848 resulted in robust IFN-β production, with significantly higher levels associated with DC *Ndst1* mutation. In vivo, improved pathogen clearance in *Ndst1f*/f *CD11cCre* + mutant mice was suggested by reduced IAV AA5H nucleoprotein in lung examined in the late/effector phase. Earlier in the course of infection (day 5 p.i.), mean viral load, as measured by viral RNA, was not significantly different among genotypes. These findings point to novel regulatory roles for DC HS in innate and adaptive immunity during viral infection. This may have therapeutic potential and guide DC targeted HS engineering platforms in the setting of IAV or other respiratory viruses.

## Introduction

Global mortality from influenza has historically affected hundreds of thousands of lives yearly, and remains a serious ongoing and future threat. The dominant types causing seasonal epidemics of disease are influenza A and B viruses (IAV and IBV)^1^. In IAV, nuclear export protein and a segmented RNA genome coated with nucleoprotein (NP) and RNA-dependent RNA polymerase are enclosed within the matrix protein layer. The envelope anchored hemagglutinins, neuraminidases, and matrix ion channels facilitate host cell entry^2^. During an IAV infection, airway macrophages secrete inflammatory cytokines in a manner that may eliminate IAV and its components. If unregulated, intense inflammatory cytokine release may contribute to poor clinical outcomes by inducing airway congestion, impaired gas exchange, secondary bacterial pneumonia, and acute respiratory distress syndrome with its complications^3^. On the other hand, a balance of cellular influx and effector mechanisms are important for viral clearance and acquired immunity.

Pathogen clearance and immunomodulation can be harmonized by antigen presenting cells, including dendritic cells (DCs) that orchestrate both innate and adaptive immunity. Following exposure to IAV components or phagocytosis of IAV-infected cells, myeloid DCs may recognize components of the IAV genome and other pathogen and damage associated motifs through endosomal toll like receptor (TLR) pathways^4^. Sensing of viral infection additionally occurs through TLR3, retinoic acid-inducible gene I (RIG-I)-like receptors (RLRs), and TLR7 (recognizing IAV ssRNA), among others; and this results in activation of both downstream pro-inflammatory signaling events along with expression of type-I interferons^4–6^. Even exogenous stimulation of other TLR pathways (e.g., TLR9 via exogenous CpG) have been shown to facilitate DC activation and anti-IAV responses^7^, although lung DCs may theoretically be less responsive than myeloid DCs (based on TLR9 expression) to such exogenous stimulation^8^. Signaling by several TLRs includes downstream activation of NF-κB, which in turn upregulates antiviral cytokine, type 1 interferon (IFN-I) (α/β) expression^9^. IFN-β induces the signal transducer and activation of transcription (STAT) signaling pathway, which upregulates IFN stimulated genes that inhibit viral replication within infected cells^10^. Additionally, IFN-β recruits natural killer (NK) cells to eliminate IAV infected cells at the site of infection^11^. Cross-talk between NK cells and DCs also promotes lymphoid-directed DC migration and initiation of IAV-specific T cell immunity by IAV antigen presenting DCs^12^.

The functions of DCs may be tuned by the structure of cell surface glycosaminoglycans (GAGs). GAGs are linear polysaccharides that are generally sulfated and linked to proteoglycan (PG) core proteins; and are involved in various biological processes including cell adhesion, signaling transduction, and pathogen recognition^13–15^. In recent work, we showed that a DC-directed genetic alteration of heparan sulfate (HS) GAG enhances antigen/MHC-I (major histocompatibility complex) presentation and cytotoxic T cell responses in tumor bearing *Ndst1f/f CD11cCre*^+^ mutant mice compared to wildtype (*Ndst1f/f CD11cCre*−) control mice^16^. N-deacetylase/N-sulfotransferase (Ndst1) is a bifunctional enzyme that catalyzes N-deacetylation and the N-sulfation of glucosamine during modification steps involved in the biosynthesis of nascent HS glycan chains in the Golgi complex^13^. Utilization of the *CD11c Cre* promoter results in efficient deletion in conventional DCs, and also some monocyte/macrophages and NK cell populations in mice^17^, which could result in altered HS in both DCs as well as alveolar macrophages in the lung. Deletion of Ndst1 in macrophages (which has been demonstrated in *Ndst1f/f LysMCre* + mutants)^18^ interestingly leads to upregulation of IFN-β expression. Moreover, deficiency in syndecan-4, a major DC-surface HSPG, results in a more enriched NK cell population in tumors^19^.

In addition to a possible mechanism involving “boosting” of IAV specific antigen presentation to responsive T cells in the setting of under-sulfated HS on DCs, these findings prompted us to query whether targeting the fine structure of DC HS might also regulate IFN-β, and augment IAV-clearance while modulating the level of inflammation during IAV infection. In the current study, we phenotypically examined the degree of inflammation along with unique tissue T-cell responses, while functionally characterizing IAV-nucleoprotein levels in the lungs of IAV-infected *Ndst1f/f CD11cCre* + mutant and *Cre*− wildtype control mice examined in the early and late IAV infection phases. Using Ndst1 knock-down in a model DC line DC2.4, we investigated mechanistic details including NF-κB p65 subunit phosphorylation and IFN-β expression by quantitative PCR (qPCR) in response to a model TLR9 ligand, CpG. We extended studies to primary marrow-derived DCs, wherein we assessed IFN-β protein responses to CpG and to an IAV relevant TLR7 ligand. Lastly, CD4^+^/FOXP3^+^ regulatory T cell (T_reg_) and CD8^+^ T cell phenotypes were examined in lungs of IAV-infected wildtype and mutant mice.

## Results

### A CD11c + cell directed Ndst1 mutation reduces IAV-associated lung inflammation in mice in the effector phase

**Figure 1:**
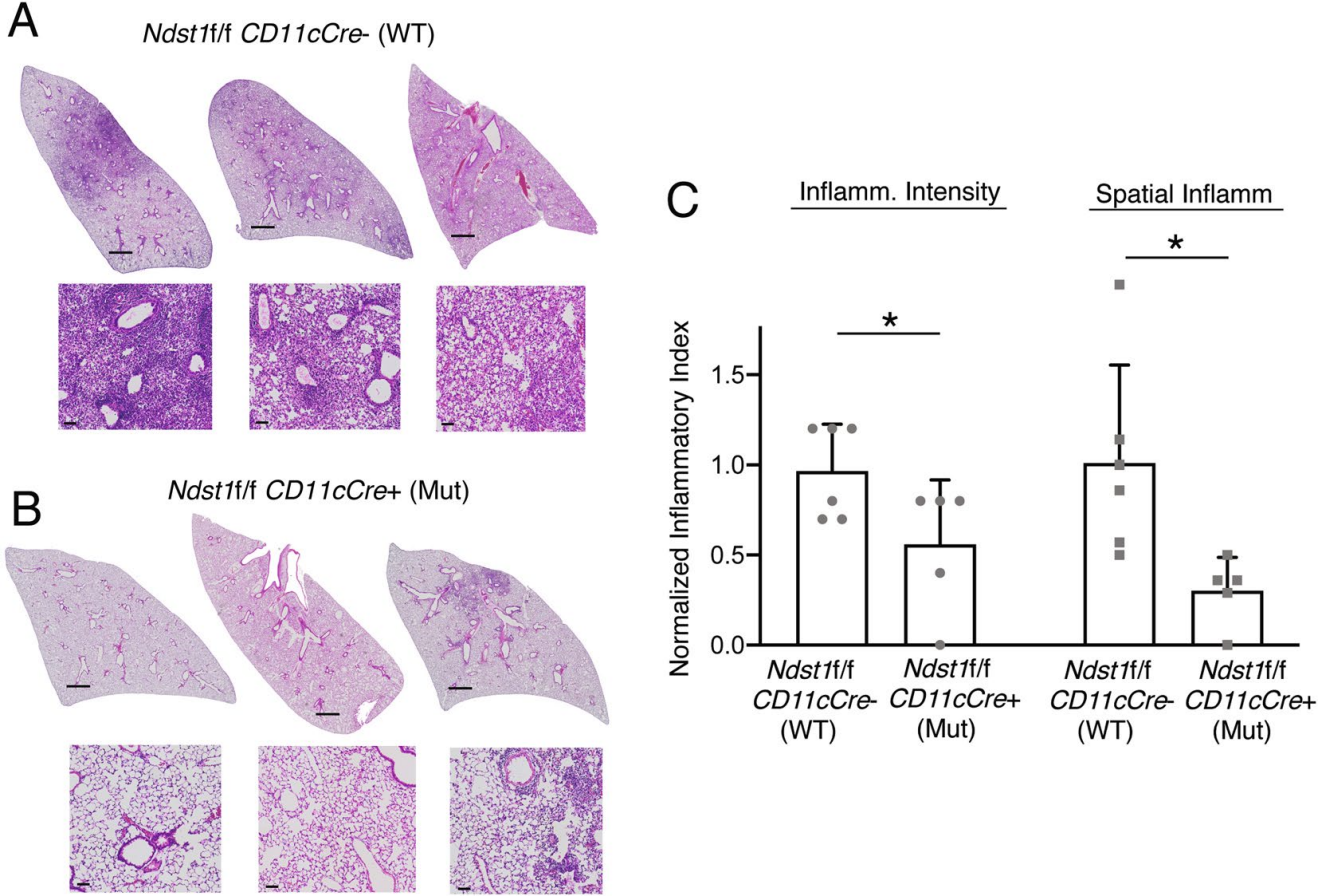
The magnitude of IAV lung inflammation is reduced in *Ndst1f*/f *CD11cCre* + mutant mice, with inhibition in inflammation compared to that in *Ndst1f*/f *CD11cCre*− wildtype controls at day 9 post-inoculation. Mutant and wildtype mice were inoculated with an intranasal dose of 20 PFU of PR8 IAV (*n* = 5 mutant and n = 6 wildtype). At day 9 post-inoculation (p.i.) lungs were harvested, inflated, paraffin embedded, and sectioned. (**A**) Representative images of H&E-stained lung sections from IAV-infected *Ndst1f*/f *CD11cCre*− wildtype mice are shown, with representative 400X photomicrographs from inflammatory zones shown below each section (scale bar = 1 mm for low-power whole-lung images; bar = 50 μm for 400X photomicrographs). (**B**) Images of H&E-stained lung sections and representative photomicrographs from IAV-infected *Ndst1f*/f *CD11cCre* + mutant mice. (**C**) Histopathology from lung sections was analyzed by a trained pathologist blinded to genotype; and mean inflammatory index for each lung (estimated on a 0–3 scale of severity by the pathologist) was normalized from inflammatory scores of inflamed areas on lung sections. Inflammatory indices were assessed through analyses of both pathologic intensity of inflammation (left) as well as the mean spatial inflammatory involvement of lung sections (right). Spatial inflammatory involvement for any given lung section (percentage of section with any inflammation) was also initially determined through quantification by counting blinded to genotype. Mean +/− SD for the measures normalized to wildtype is shown on the graph (*P = 0.03 and *P = 0.02 for differences in means for intensity and spatial measures, respectively).

While inflammation is a necessary part of the innate immune response upon IAV infection, it can result in morbidity and mortality through airway congestion, impaired gas exchange, acute respiratory distress syndrome, and as a substrate for secondary bacterial pneumonia if unchecked^3^. To determine the level of inflammation in the lungs of IAV-infected wildtype *Ndst1f*/f *CD11cCre* − and mutant *Ndst1f*/f *CD11cCre* + mice, histopathology specimens were reviewed by a trained pathologist blinded to genotype. Hematoxylin and eosin (H&E)-stained paraffin-embedded sections were prepared from the lungs of mice 5- and 9-days post-inoculation (p.i.) with IAV, examining the early-infection and late/effector phases, respectively^20^. Indices of inflammatory intensity, determined from the most inflamed zone of each lung section, as well as spatial inflammation (as a percentage of total lung area on whole-lung histologic sections) were then determined. H&E-stained lung sections harvested on day 9 p.i. demonstrated less infiltration of inflammatory cells into a predominantly airway-centered distribution of inflammation in the mutant mice compared to that of wildtype mice (Fig. 1A,B). We noted approximately 50% and 70% reductions in inflammation intensity and spatial inflammation, respectively, in the lungs of mutant mice compared to those of wildtype mice (from n = 5 mutant and n = 6 wildtype mice; P < 0.05 for differences) (Fig. 1C). In mice for which lung sections were harvested at 5 days p.i. there were no significant differences in inflammatory intensity and spatial inflammation between wildtype and mutant mice (from n = 3/genotype; Supplemental Fig.S1). A summary of histologic inflammation scoring, including mean values +/− SD, that tabulates data from Fig. 1 and Supplemental Fig.S1 (for studies at day 9 and day 5 p.i.) is presented in Supplemental Table 1. Weights of wildtype and mutant mice were tracked throughout the course post-inoculation, and plotted in Supplemental Fig.S2. Interestingly, while there were no significant differences in mouse weight trends through the early course post-inoculation, final weight analyses at day 9 p.i. showed that among two experiments (n = 10 mice/genotype), 7/10 wildtype mice demonstrated > 10% weight reduction relative to baseline, while only 3/10 mutants showed such a reduction (P = 0.07 for difference by Chi-square statistic). Moreover, the mean % weight reduction beyond 10% was significantly greater in wildtype mice compared to that in mutants (5.6% versus 2.9%; P < 0.001 for difference).

### Ndst1 silencing enhances CpG-dependent NF-κB phosphorylation in DC2.4 cells

**Figure 2:**
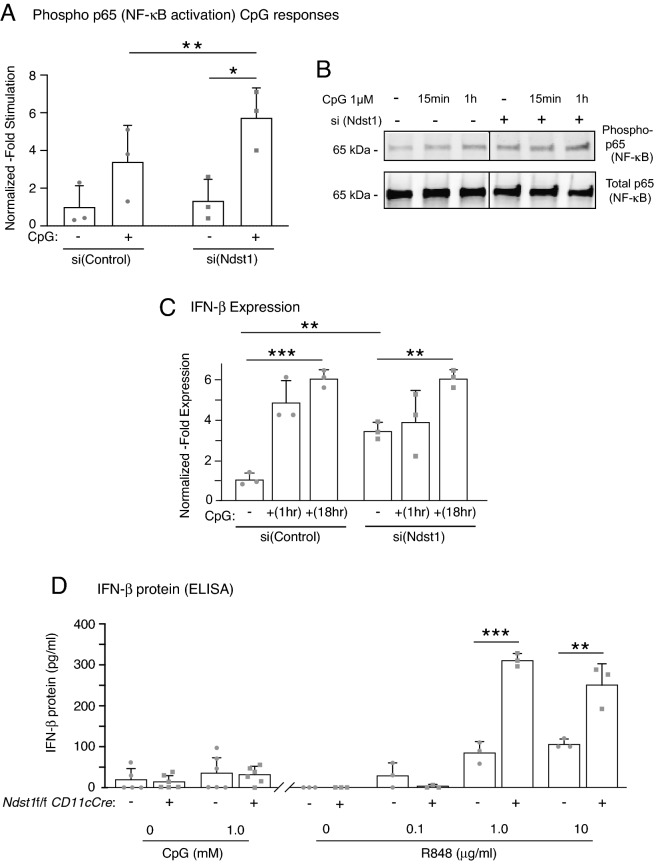
Ndst1 silencing results in increased NFκB activation in response to CpG and interferon-β expression in DC2.4 cells as well as augmented interferon-β protein production in primary *Ndst1* mutant DCs stimulated by the TLR7 ligand R848. (**A**) CpG-dependent NFκB activation is significantly augmented by Ndst1 silencing of DC2.4 cells, designated si(Ndst1), compared to that of si(Control) DC2.4 cells (i.e., scrambled RNA treated controls). Specifically, the level of phosphorylated NFκB subunit p65, as measured by western blotting, was indexed to the corresponding total p65 value; and the graphed values for each condition (at baseline and at 1 h post stimulation) are shown normalized to that of un-stimulated si(Control) cells in the graph (mean of n = 3 experiments, *P = 0.03 and **P < 0.01 for indicated differences in means; paired T-test). (**B**) A representative Western blot from one NF-κB activation experiment is shown to the right of the graph in (**A**), illustrating bands for phospho-p65 and total p65 collected from DC2.4 cells stimulated with 1 μM CpG for 15 min and 1 h (with data for baseline and 1 h stimulation plotted in the graph); and stimulation in the absence or presence of Ndst1 silencing denoted by “–” or “ + ” above each lane (full length blot shown in Supplemental Fig. S8). (**C**) Interferon-β quantitative PCR results for basal/unstimulated DC2.4 cells as well as cells stimulated with CpG. Among unstimulated cells: Ndst1 silenced DC2.4 cells demonstrate a significant increase in interferon-β expression (IFN-β) relative to that of control cells. Percent IFN-β expression was normalized to GAPDH expression in the respective cells, and responses for each condition are normalized to the response by resting si(Control) DC2.4 cells. Graph shows mean +/− SD from triplicate runs; **P < 0.01 for difference in means for si(Control) versus si(Ndst1) cells. Stimulation with CpG showed increased IFN-β expression responses by both control and Ndst1-silenced cells (to 1 μM CpG). While expression responses to 18 h CpG exposure were significant compared to baseline for both si(Control) and si(Ndst1) cells (***P < 0.001 or **P < 0.01 for respective differences in means relative to baseline), they reached essentially equivalent expression levels by the 18 h exposure time point (right bars on Fig. 2C graph). (**D**) IFN-β protein production into the culture medium was examined by ELISA assay in primary *Ndst1f*/f *CD11cCre* + mutant or *Ndst1f*/f *CD11cCre*− control bone marrow DCs in response to either (i) presence versus absence of 1 μM CpG stimulation overnight (high dose/ maximum stimulation from prior studies); or (ii) presence (up to 10 μg/ml) versus absence of overnight stimulation with the TLR7 agonist R848. While we were able to measure only minimal IFN-β protein responses upon CpG stimulation, with no significant differences compared to basal/resting cells (graph portion at left), primary DCs responded with significant IFN-β production in response to R848 at the 1.0 or 10 μg/ml range, with markedly greater responses associated with DC *Ndst1* mutation (right portion of graph; ***P < 0.001 and **P < 0.01 for differences in indicated mean values).

DCs may sense the CpG dinucleotide motif as a model TLR9 ligand that leads to classical downstream TLR signaling events including phosphorylation of IκBα by IKK complex, translocation of liberated NFκB into the nucleus, and initiation of IFN-β transcription^4,7^. Phophorylation of NFκB impacts transcriptional activity of NFκB through modulating transcription of target genes and other transcriptional factors^21^. Phosphorylation of NFκB p65 subunit in particular leads to these functional changes by induction of a conformational change, p65 ubiquitination and stability, and interactions with other factors^21^. We determined the levels of phosphorylated p65 (phosphorylation at S536) and total p65 protein in control and Ndst1 silenced DC2.4 cells treated with and without CpG by western blotting. Control and Ndst1 silenced DC2.4 cells were serum starved for one hour prior to CpG stimulation to mimic the nutrient deprived condition during viral replication. We demonstrated that Ndst1 inhibition in DC2.4 cells significantly augments CpG-dependent NFκB phosphorylation (approximately 1.7 -fold) compared to that of control DC2.4 cells (P < 0.01 for difference; Fig. 2A,B). Similar patterns were shown in non-starved cells (not shown), although the relative degree of augmentation in CpG responses by mutant cells was greater under serum-starved conditions.

### Ndst1 silencing results in increased basal interferon-β expression in DC2.4 cells

IFN-β is an antiviral cytokine that inhibits viral replication within an infected cell and recruits NK cells to the infection site to eliminate IAV-infected cells^10,11^. We transfected DC2.4 cells with vehicle (scrambled siRNA) control, “si(Control)” and Ndst1 siRNA, “si(Ndst1),” and we examined basal levels of IFN-β expression by qPCR. DC2.4 cells are immortalized DCs generated by transducing bone marrow isolates of C57BL/6 mice with retrovirus vectors expressing murine granulocyte–macrophage colony-stimulating factor, and the *myc* and *raf* oncogenes^22^. IFN-β copy number normalized to glyceraldehyde 3-phosphate dehydrogenase (GAPDH) demonstrated about four-fold increase in IFN-β expression in Ndst1 silenced DC2.4 cells in comparison with that of control cells (Fig. 2C, left bars corresponding to basal un-stimulated cells; P < 0.01 for difference in means). Ndst1 deletion efficiency in the cell line is shown in Supplemental Fig.S3. In a separate assay, DC2.4 cells were stimulated in culture using CpG, and while expression of IFN-β was significantly increased by CpG stimulation in both Ndst1 silenced DC2.4 cells and control cells by qPCR (P < 0.01 for mean expression responses relative to control; Fig. 2C), we could not reproducibly detect increases in IFN-β protein in cell lysates by western, although low levels without significant differences were detected by ELISA assay in the culture cell medium (Fig. 2D, left side of graph).

### Interferon-β protein response to TLR7 stimulation is augmented in Ndst1 deficient primary bone marrow DCs

We next examined IFN-β protein production by primary bone marrow DCs (BMDCs) purified from *Ndst1f*/f *CD11cCre* + mutant and *Cre*− wildtype mice. While we were unable to detect significant increases in IFN-β protein in either cell lysates or culture supernatant (by Western or ELISA methods) to the TLR9 ligand CpG upon overnight exposure, primary DC responses to the TLR7 ligand resiquimod (R848) resulted in IFN-β production, with significantly greater levels associated with DC *Ndst1* mutation (Fig. 2D). In particular, *Ndst1f*/f *CD11cCre* + mutant DCs exposed to 24 h stimulation with 1 or 10 μg/ml of R848 generated a marked increase in IFN-β production into the culture medium relative to that of *Cre*− wildtype control cells (P < 0.01 for difference using either 1 or 10 μg/ml R848 concentration). The maximum response by either mutant or wildtype DCs under the culture conditions used appeared to take place at 1 μg/ml R848, with > threefold response by mutant over that of control cells at that concentration (Fig. 2D, right). We assayed whether for R848 stimulation there might be differences in NFkB responsiveness by mutant and wiltype DCs, and did not note a significant difference in p65 subunit phosphorylation in short-term stimulation assays at 15 min and 1 h post-stimulation (Supplemental Fig.S4).

### CD11c + cell directed Ndst1 mutation is associated with increased pathogen clearance in lungs of IAV-infected mutant mice in the effector phase

**Figure 3:**
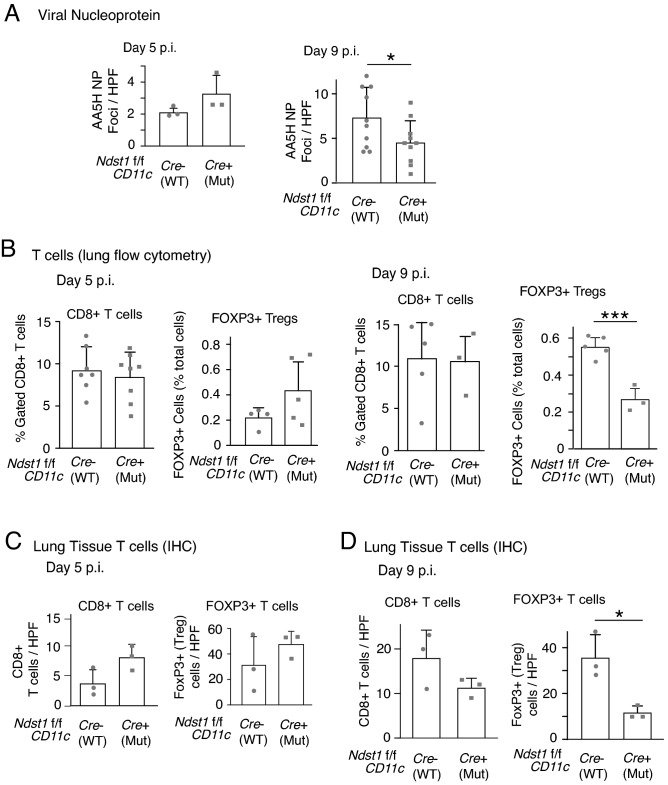
Reduced IAV AA5H nucleoprotein and a lower density of regulatory T cells is found in lung tissue of IAV-infected *Ndst1f*/f *CD11cCre* + mutant mice at day 9 post IAV-inoculation. Lungs from mutant and wildtype animals were harvested, inflated, paraffin embedded, and sectioned, with IHC performed using antibodies specific for IAV AA5H nucleoprotein (NP), with assessments for lung CD8 + T cells, and FOXP3 + T regulatory cells (T_regs_) as illustrated. Measurements of AA5H NP density in tissue by IHC as well as T cell densities were carried out blinded to genotype. (**A**) To assess trends reflecting the early course of infection, analysis of lung sections from *Ndst1f*/f *CD11cCre* + mutant versus *Cre*− wildtype mice was carried out at day 5 p.i. (n = 3 mice per genotype): Mean AA5H viral NP deposits per high power field (HPF) in lung IHC sections +/− SD are plotted in graph at left. No significant difference was found between mean values for groups at day 5 p.i. On the other hand, *Ndst1f*/f *CD11cCre* + mutant mouse lungs at day 9 p.i. exhibited reduced pathogen presence, as reflected by reduced mean AA5H viral NP deposits per HPF (right graph; with data from n = 10 mice/genotype; *P < 0.05 for difference in means). (**B**) T cell flow cytometry was carried out to assess the density of CD8 + T cells (% gated from total lung-cell digest) and FOXP3 + T_reg_ cells (as % total lung cells) in whole-lung digests from mutant and wildtype mice at either day 5 p.i. (left graphs) or day 9 p.i. (right graphs) in separate experiments. At day 5 p.i., there were no significant differences between genotypes for either the lung CD8 + T cell density (from studies involving a total of n = 7 wildtype and n = 8 mutant mice) or lung T_reg_ cells (n = 4 wildtype and n = 5 mutant mice); although P = 0.07 was noted for difference in T_reg_ mean values (greater mean T_reg_ density noted in mutant lungs) at that timepoint. At day 9 p.i., while there were no significant differences in mean CD8 + lung T cells, there was a significant reduction in FOXP3 + T_reg_ cells in lungs from mutant mice (n = 5 wildtype and n = 3 mutant mice; ***P < 0.001 for difference in means). (**C**) In IHC analyses of T cells in lung tissue at day 5 p.i., the mean density of CD8 + T cells per HPF (left graph) as well as that of FOXP3 + T_reg_ cells (right) showed no significant differences in mean values for each genotype (n = 3 mice per genotype). (**D**) In IHC analyses of lung T cells at day 9 p.i., the mean density of CD8 + T cells was not significantly different among genotypes; however, the density of FOXP3 + T_reg_ cells in the lungs of *Ndst1f*/f *CD11cCre* + mutant mice at day 9 p.i. was significantly reduced compared to that of *Cre*− control lung tissue sections (right graph; *P = 0.02 for difference in means; n = 3 mice per genotype).

While augmented T-cell mediated immunity induced by DCs may promote effector responses to facilitate viral clearance in the late/effector phase of infection by IAV, one of the consequences of upregulated IFN-β responses by *Ndst1* mutant DCs may also be inhibition of viral replication. To initially estimate the effect of *Ndst1* mutation on viral presence and pathogen clearance, we quantified the amount of IAV nucleoprotein (NP) present in the lungs of IAV-infected wildtype and mutant mice harvested on day 5 and day 9 p.i. by performing immunohistochemistry (IHC). The IAV NP is a structural protein that encapsulates the viral RNA within the matrix protein layer^2^, and can be used to assess viral presence in tissue. While there was no significant difference between wildtype and mutant in the levels of AA5H IAV NP on day 5 p.i (Fig. 3A, left) (also carried out separately by flow cytometry in Supplemental Fig. S8), we observed almost 40% reduction in the AA5H IAV nucleoprotein in the lungs of IAV-infected mutant mice in comparison with that of wildtype mice at day 9d p.i. (P < 0.05 for the difference in means; Fig. 3A, right). Representative images of IHC-stained lung sections of IAV-infected wildtype and mutant mice at day 9 p.i. are provided in Supplemental Fig.S5. Since IHC studies of viral NP may have limitations in terms of the ability to accurately reflect levels of prior replicating virus in the lung tissue, we probed for the potential to detect replicative virus in lung harvested from mice at the earlier (day 5 p.i.) time point. While virus was not detectable in the tissue by plaque assay, we were able to detect viral RNA by qPCR using primers for the IAV M1/M2 protein^23^, and additionally upon using primers for PR8 NP^24^ in separate measurements. Both detection methods yielded similar mean levels of viral RNA from lungs of mutant versus wildtype mice at day 5 post-inoculation (i.e., 5 +/− 6 × 10^6^ versus 4 +/− 6 × 10^6^ PFU per μg lung cDNA for viral RNA purified from whole mutant (n = 6) versus wildtype (n = 4) lungs; and with the PFU equivalent values referenced from a standard curve generated from qPCR cycle-thresholds corresponding to known dilutions of RNA from pure PR8 IAV at a known starting viral titer). It is noteworthy, however, that lungs from 3 of the n = 6 *Ndst1f*/f *CD11cCre* + mutant mice had no detectable viral RNA by qPCR at the day-5 timepoint (albeit with widely variable levels in the other 3 mutants), while lungs from all n = 4 *Cre*− wildtype mice harbored some level of detectable virus. We thus cannot be certain if this reflects earlier viral clearance in those mutant mice, but this additional observation raises a possibility. We present qPCR data showing cycle thresholds corresponding to expression of IAV M1/M2 protein in lung tissue for each mouse and the referenced standard curve data (showing qPCR cycle thresholds corresponding to known dilutions of RNA from a pure PR8 IAV stock at a known starting viral titer) in Supplemental Table 2 for reference.

### Lung T cell responses during IAV infection demonstrate a late/effector phase T_reg_ cell phenotype in the setting of Ndst1f/f CD11cCre + mutation

While a CD4 + subset of T_reg_ cells may induce immune-suppressive functions in the setting of tumor or pathogen responses, T_reg_ cells may also provide a protective role in the setting of pulmonary damage during viral infection^25^. Along the lines of the latter observation, T_reg_ cells have been implicated in recovery from IAV induced inflammation (including enhancement of germinal center responses)^26,27^. In excess, however, T_reg_ cells can suppress cytotoxic functions of effector CD8^+^ T cells that eliminate IAV-infected cells^28^. In early immunophenotyping, we ran flow cytometry to quantify CD8 + T cells and FOXP3 + T_reg_ subsets on whole-lung digests from mice at day 5 p.i. Quantification of CD8 + T cells in the lungs was comparable among genotypes at this time point (Fig. 3B, left CD8 + T cell graph); and the mean densities of T_reg_ cells in the lung at this time point (Fig. 3B, left FOXP3 + Tregs graph) also did not show statistically significant difference in means between genotypes (although for T_reg_ cells, *P* = 0.07 for the difference, with a greater mean value found in *Ndst1f*/f *CD11cCre* + mutants relative to that of *Cre*− controls). There were also no significant differences in total CD4 + T cells from mutant versus wildtype lungs (Supplemental Fig.S6). On the other hand at day 9 p.i., while there were no significant differences in mean CD8 + lung T cells among genotypes, there was a significant reduction in FOXP3 + T_reg_ cells in lungs from mutant mice (*P* < 0.001 for difference in means). Through IHC T-cell analyses on paraffin-embedded lung sections of IAV-infected wildtype and mutant mice harvested on days 5 and 9 p.i., we characterized and quantified CD8 + T cells and CD4^+^/FOXP3^+^ T_reg_ cells in lung tissue, in situ. While no significant difference in T_reg_ cell density was noted at day 5 p.i. (noting a repeated trend toward greater T_regs_ in mutant lungs at that time point; Fig. 3C, right graph), lungs examined in a separate study carried out to day 9 p.i. were characterized by a density of lung tissue T_reg_ cells that was significantly lower in mutant mice at this late/effector phase time point (P < 0.05 for difference in means; Fig. 3D, right), reflecting findings by flow cytometry at this time point (Fig. 3B, right). In tissue CD8 + T cell analyses, mutation was not associated with significant differences in CD8 + T cells at day 5 or day 9 p.i. (Fig. 3C, left and Fig. 3D, left).

## Discussion

Balance between pathogen elimination and immunomodulation is critical for survival outcomes during IAV infection^3^. Based on our previous findings, we hypothesized that a DC-directed Ndst1 mutation may boost pathogen clearance and favorably modulate inflammation in IAV-infected mice. In the current study, we demonstrate that IAV-infected *Ndst1f*/f *CD11cCre* + mutant mice exhibit significantly reduced inflammation in the lungs compared to those of IAV-infected *Ndst1f*/f *CD11cCre*− wildtype mice in the effector phase (day 9 p.i.) while there was no significant difference between wildtype and mutant mice in the early infection phase (day 5 p.i.) (Fig. 1A–C; Supplemental Fig.S1). The reduced inflammatory phenotype found at day 9 p.i. may be partly due to a more rapid/efficient cycle of early effector activity and ultimate immunologic resolution (and possibly augmented DC-mediated antigen presentation) and downstream effector T cell responses in the setting of under-sulfated DC HS. This is reminiscent and consistent with antigen-mediated responses we have noted in the setting of the same mutation during anti-tumor responses in neoplasia models^16^. However, unique effects on TLR dependent DC signaling and associated anti-viral effector pathways must also be considered.

Possibly, the reduced inflammatory phenotype may also be due to upregulated expression of the antiviral cytokine IFN-β, and may be further supported by enhanced TLR-dependent NF-kB activation, as demonstrated through CpG-dependent NF-κB p65 subunit phosphorylation in Ndst1 silenced DCs (Fig. 2A,B). Phosphorylation of NF-κB p65 subunit leads to a conformational change influencing p65 ubiquitination, stability, and interactions with its ligands^21^. IFN-β activates the STAT signaling pathway, leading to expression of IFN stimulated genes that inhibit viral replication within infected cells and other downstream signaling components including interleukin-10 (IL-10)^10^. While we measured a boost in IFN-β expression in vitro in resting Ndst1-silenced DC2.4 cells (Fig. 2C, compare expression in control-to that of Ndst1 silenced basal/un-stimulated cells), in vivo cell-specific measurements and tissue levels may be of interest because other myeloid cells, including macrophages, have demonstrated some degree of conditional allele deletion in *CD11cCre* targeted mice^16,17^. Although it is beyond the scope of this study, this may be carried out in future studies examining broader production of the type-I IFN family by glycan-targeted antigen presenting cells in-situ, possibly in advanced in-tissue studies. Another study reported elevated levels of IL-10 expression in bone marrow derived macrophages from *Ndst1f*/f *LysMCre* + mice compared to those from wildtype *Cre*− controls^18^, indicating that reduced inflammation may be at least partly achieved through inhibition in macrophage activation^29^, and possibly a protective effect against lung damage that may be induced by optimizing levels of type I IFN, while recognizing that excessive type I IFN expression in the lung may be detrimental^30,31^. If the mutation augments protective role(s) by macrophages, known to exist in the setting of IAV infection^32^, given additional possible effects on macrophages in the setting of CD11c driven *Ndst1* mutation, this might also be considered a protective factor in this unique phenotype. In the prior study^18^, Ndst1 deficient macrophages appear to release IFN-β, which is typically regulated by binding to wildtype fully-sulfated HS (thus sequestered by the fully sulfated glycan). Mutation appears to release IFN-β for greater action/availability to activate downstream STAT1 signaling in the macrophage^18^. Possibly similar mechanisms may occur in parallel in both DCs and macrophages (including alveolar macrophages) in the setting of *Ndst1* mutation. Indeed greater IFN-β production/release into the medium may also be a consequence of inhibiting HS-dependent sequestration in the setting of HS mutation/under-sulfation when IFN-β production/stimulation takes place (e.g., with robust TLR7 dependent IFN-β stimulation in the setting of mutation; Fig. 2D). Finally, while preliminary studies of NK cell density in the lungs of *Ndst1f*/f *CD11cCre* + mutant mice harvested on day 5 p.i. did not show a significant difference from that of wildtype mice (data not shown), it is of interest to further investigate the potential regulatory role of HS in DC-NK cell crosstalk in the priming of immune responses to IAV infection in future studies.

Interestingly, we observed a reduction in IAV viral particles, as measured by AA5H NP in the lungs of mutant mice compared to those of wildtype mice at day 9 p.i., However no significant difference was shown on day 5 p.i. (Fig. 3A). In the latter studies, carried out to examine an earlier timepoint post-inoculation, we also quantified the AA5H viral NP in mutant and widtype tissues by flow cytometry (Supplemental Fig.S7). From these findings, and from additional measurements of viral RNA by qPCR at day 5 p.i. in separate studies reported in the Results section, DC associated *Ndst1* mutation does not appear to have a profound effect on inhibiting viral replication in the early infection phase, while the mutation may possibly augment IAV clearance in the late infection/effector phase. In terms of NP detection in IHC studies, we also recognize that while NP presence in IHC tissue is alone not sufficient to reflect viral replicative potential in tissue at a given time point, the NP IHC studies may reflect the prior presence of virus. It is thus possible that a low level of viral replication that persists in the wildtype in the day 5–6 dpi period and slightly beyond (which could leave a greater density of IHC positive NP “remnant” protein in the tissue by day 9) may result in comparatively lower NP positive material in the mutant due to increased viral clearance taking place over that same period of fading viral replication in the protocol IAV model.

The mutant phenotype may be partly explained by upregulated IFN-β production and/or increased NF-κB activation, as shown in *Ndst1* silenced DCs under distinct assays using TLR7 or TLR9 ligand stimuli (Fig. 2), and associated with an increased DC-maturation phenotype^33,34^. We have observed this phenotype in mutant DCs in prior work, where cultured *Ndst1* deficient primary DCs more frequently showed greater expression of maturation markers CD86/MHC-II^16^. Type I IFN also modulates an ability of DCs to cross-present to CD8^+^ T cells^35,36^. This may also be mechanistically relevant to a recent finding that *Ndst1* mutant DCs possess enhanced antigen presentation capability^16^. With this in mind, it should also be noted that IAV-infected DCs become less capable of presenting IAV-antigen, resulting in impaired CD8^+^ cytotoxic T cell responses^9,37,38^. Possibly, the mutation may thus be important in limiting the effects of this vulnerability in the setting of IAV infection. Perhaps an increase in IFN-β expression may also facilitate pathogen clearance by cross-presentation mediated cytotoxic T cell immunity rather than limiting intracellular viral replication at the infection site in mutant mice.

With specific regard to IFN-β, we could demonstrate low-level IFN-β expression by model DCs in culture that respond to CpG stimulation. While responses by mutant and wildtype DCs appear to be comparable, *Ndst1f*/f *CD11cCre* + mutant DCs demonstrate augmented basal/resting IFN-β expression by qPCR. For primary DCs isolated from mutant and wildtype mice (and for DC2.4 cells), this did not translate into robust protein responses in terms of IFN-β protein produced into the culture medium, as measured by a sensitive ELISA based method. However, upon testing primary DC responses to an IAV relevant TLR7 ligand (R848), *Ndst1* mutation resulted in significant and robust increases in IFN-β protein into the medium (Fig. 2D). This is important since increased responses by mutant DCs to TLR7 stimulation, for which the natural ligand is IAV ssRNA^6^, would imply that IFN-β mediated anti-IAV responses may potentially play a relevant mechanistic role in facilitating IAV clearance during active infection in the mutant. This may also be important in facilitating inflammatory resolution. We should note that upon testing NF-κB responses to R848, measuring phospho-p65 responses to short-term (15 min and 1 h) R848 exposure in cultured primary DCs, there were no significant differences noted for mutant versus wildtype cells (Supplemental Fig. S4). It is difficult to know whether differential R848 responses by mutant versus wildtype cells in terms of overnight IFN-β production into the medium (markedly elevated in the mutant) versus short-term phospho-p65 signaling responses are due to different effects that glycan under-sulfation has on one downstream TLR7 intracellular signaling pathway (e.g., nuclear IFN-β expression via IRF7 activation) versus another (e.g., inflammatory/cytokine responses via TAK1-mediated NF-κB and MAPK pathways)^39^. Since the cultured primary cells are a predominant DC population, with some percentage of other non-cDC CD11c + subsets to a lesser extent (including up to 15–20% macrophages in our hands), it is possible that differential responses may reflect a dominant R848 effect of IFN-β production in one subset. Alternatively, among all *Ndst1* deficient CD11c + cultured primary cells, one very intriguing possibility is that dominant IFN-β release into the medium by mutant cells may result following IFN-β production, where reduced HS-mediated sequestration by *Ndst1* mutant cells may increase IFN-β release and availability, as referenced in prior studies on *Ndst1* mutant macrophages^18^, above. It is beyond the scope of this work to fully decipher these differences herein, but nevertheless, the latter consideration leaves intriguing mechanistic possibilities for mutant DCs in vivo responding to ssRNA/TLR7-ligands in the microenvironment following IAV infection.

Regulatory T cells (T_regs_) expand upon IAV infection, and are believed to suppress antiviral immunity^40,41^, while an appropriate level may be necessary to prevent excessive pulmonary inflammation and damage^25,42^. A reduction in the FOXP3^+^ T_reg_ population in the lungs of IAV infected mutant mice on day 9 p.i. (shown by flow cytometry in Fig. 3B, right and IHC in Fig. 3D, right) may potentially contribute to a boost in IAV clearance, also shown on day 9 p.i. (Fig. 3A). Interestingly, earlier in the course of infection (day 5 p.i.), there was no such reduction in lung T_reg_ cells in the mutant; and if anything, there was a trend toward augmented T_reg_ cells in mutants at this earlier phase (corresponding FOXP3 + T cell graphs in Fig. 3B, and Fig. 3C, right), which may also be important for anti-viral germinal center responses^25,27^. Strikingly, toward the resolution phase at day 9 p.i., the presence of lung T_reg_ cells is significantly reduced in the setting of mutation (in both flow cytometry and IHC studies; Fig. 3B, far right as well as Fig. 3D, right). It is also important to consider how increased IFN-β production by mutant CD11c + cells in culture might relate to increased IAV clearance. While our measurements of increased IFN-β production by mutant DCs in culture is limited to cell-based assays, it is possible that in the lung during IAV infection, this effect by the CD11c + cells in the infected lung interstitial and/or lymphoid microenvironment of mutant mice may have contributed to ultimately driving more efficient viral clearance in the mutant by the late/effector stage. Although beyond the scope of this work, dedicated studies examining the effects of antigen-presenting cell glycan targeting on IFN-β production and cell-specific effects on Treg cells in the spatial inflammatory lung microenvironment of IAV infection in the effector phase would add additional insights.

This data may reveal important antiviral phenotypes and signaling events under a DC-directed *Ndst1* silencing platform that may contribute to an effective balance of innate and acquired pathogen clearance functions, while resulting in earlier resolution in inflammation during the effector phase of viral infection. The findings and genetic target validation, along with additional translational studies, might facilitate the development of rational carbohydrate-based immunotherapeutic agents to inhibit influenza A viral lung injury; and possibly an immunologic basis for targeting IAV along with other respiratory viruses.

## Conclusions

In this study, we employed a novel CD11c directed *Ndst1 *silencing platform, and demonstrated a snapshot of how targeting DC surface HS may affect IAV infection-driven inflammation and immunity in mice. In particular, we found reduced inflammation, and what appears to be enhanced IAV clearance along with a suppressed T_reg_ phenotype in lung sections of IAV-infected mutant mice in comparison to that of wildtype mice in the late infection/effector phase. Using Ndst1 silencing in vitro, we demonstrated upregulated basal IFN-β expression, and enhanced CpG-dependent NF-κB p65 phosphorylation in *Ndst1* silenced model DCs. At the IFN-β protein level, however, while we could demonstrate only very weak responses to CpG using primary cultured DCs, responses to TLR7 stimulation were robust, and markedly increased in *Ndst1* mutant primary DCs. Our findings reveal potentially a glycan-based therapeutic potential for endogenous DC engineering or targeting to boost anti-viral immunity and limit the lung-inflammatory effects of IAV infection and possibly that of other respiratory viruses.

## Materials and methods

### Viral strain, mice, and inoculation

Mouse adopted influenza virus strain A/Puerto Rico/8/1934(H1N1) (PR8 IAV) was provided by the J. Teijaro lab, with strain as referenced^20^. Experiments were performed on 4- to 6-month-old *Ndst1f*/f *CD11cCre* + mutant and *Ndst1f*/f *CD11cCre*− control littermate mice. We employed mice 4- to 6-months of age to maintain consistency with original pilot studies that showed significant differences in inflammation between mutant and wildtype controls at that age range in a pilot cohort (while also paralleled by coincident limitations in breeding during COVID-19 pandemic conditions). Two additional day 5 post-inoculation studies were carried out as: (i) n = 3 mutant/n = 3 wildtype, under COVID-19 vivarium limitations for inflammation and initial AA5H and T cell IHC studies; and (ii) n = 6 mutant/n = 4 wildtype for additional data examining viral load (viral RNA studies) and T cell flow cytometry studies. For other data, the largest total mouse cohort for which mice were assessed for viral load at day 9 post-inoculation (p.i.) was mixed male/female n = 10 mutant (2 male/8 female) and n = 12 wildtype (11 male/1 female), split among 2 separate experiments with subsets examined in inflammation studies. Animal protocols were approved by the Institutional Animal Care and Use Committee (UC San Diego). Mice were intra-nasally inoculated with 20 plaque-forming unit (PFU) suspended in 40 μL PBS (C_f_: 0.5 PFU/μL) of PR8 IAV. Mice were monitored by body weight and activity over the infection period. The weight trends of wildtype and mutant mice during the course of infection are plotted in Supplemental Fig.S2. On Day 9 p.i., lungs were harvested, inflated, paraffin embedded, and sectioned for histology. We and others have used *Ndst1f*/f conditional (*Cre*−) mice as the essential and standard littermate controls to compare phenotype(s) to tissue-specific *Cre* + mutants (i.e., with cell-specific *Ndst1* deficiency in *Cre* + *Ndst1f*/f mutants). (We nevertheless reference a *Ndst1*^+*/*+^ *Cre*^+^ control that had been employed in cerebral development studies examining *Ndst1* conditional mutants, and where a wildtype *Ndst1*^+*/*+^ *Cre*^+^ state was used to demonstrate normal *Ndst1* expression in mouse cerebral tissue^43,44^.) All methods were performed in accordance with the relevant *Scientific Reports* guidelines and regulations, and this includes compliance with ARRIVE guidelines as well as American Veterinary Medical Association (AVMA) guidelines. To quantify lung inflammation, the criteria for measurement of the intensity of inflammation were the application of a possible range from 0 (min) to 3 (max) on relative intensity scale used for estimating degree of inflammation on H&E stained slides by a Pathologist blinded to genotype (whole-lung slide for each mouse). Measurement of the spatial extent of lung inflammation (% area covered) on lung histologic sections was also determined by a Pathologist blinded to genotype (whole-lung slide for each mouse). Values were normalized for graphs (Fig. 1 and Supplemental Fig.S1) to the mean value for wildtype (*Ndst1f*/f *CD11cCre*−) control mice. The values, including normalized data values, are summarized in Supplemental Table 1 as well.

### Histology

Hematoxylin and eosin staining (H&E) staining was performed on some slides. Slides for antibody labeling were prepared by deparaffinization, re-hydration, and heated citrate antigen retrieval. Avidin/Biotin Blocking Kit (Vector) was used to minimize non-specific binding in some studies using biotinylated antibody. Anti-IAV Nucleoprotein AA5H (Abcam) (1:1000 dilution) staining was performed using the M.O.M. Kit per manufacturer instructions (Vector). Anti-FOXP3 (Invitrogen) (1:100 dilution) labeling was carried out in 1% BSA/TTBS solution overnight at 4˚C. Anti-Rabbit Biotin (Vector) at 1:100 dilution in 1% BSA/TTBS solution was added to the tissue sections at room temperature for 1 h. For CD8 staining, anti-Mouse CD8 Biotin (Invitrogen) (1:100 dilution) labeling was carried out in 1% BSA/TTBS solution overnight at 4˚C. Alkaline Phosphatase Streptavidin and Vector Blue Substrate Kits were used for detection (Vector). Quantification of AA5H nucleoprotein as well as T cells in IHC tissue sections was carried out by counting of 5 high power fields (HPF) per histologic section through whole lung by two independent technical associates blinded to slide information.

### Cell culture

The bone marrow dendritic cell line DC2.4 (Millipore-Sigma) was cultured in growth medium (RPMI-1640 (Gibco) supplemented with 10% heat-inactivated FBS, 1 mM nonessential amino acids, 2 mM L-glutamine, 10 mM HEPES, 55 μM beta-mercaptoethanol, 100 U/ml penicillin, and 100 μg/ml streptomycin) at 37 °C and 5% CO_2_. Primary mouse bone marrow dendritc cells (BMDCs) were derived by harvesting whole bone marrow from either *Ndst1f*/f *CD11cCre* + mutant mice or *Ndst1f*/f *CD11cCre*− wildtype controls into cell culture, and differentiating in the presence of GM-CSF for a total of 9 days. For some studies, on day 7 of differentiation, the cells were split into a 12 well dish with 1 mL of GM-CSF supplemented media.

### siRNA transfection

Adherent DC2.4 cells at 70% confluency were incubated with transfection mixture containing Ndst1 siRNA (C_f_: 20 nM) (Integrated DNA technologies) lipofectamine RNAiMax (Invitrogen) in Opti-MEM at 37 °C for 6 h. NC1 (scrambled) siRNA was used as a negative vehicle control. Upon removal of transfection mixture, cells were maintained in growth medium overnight at 37 °C and 5% CO_2_ until use.

### NFκB phosphorylation and TLR ligand stimulation

Ndst1 silenced and non-silenced DC2.4 cells were incubated in serum free RPMI-1640 for 1 h, and then treated with 1 μM Class B CpG oligonucleotide (InvivoGen) in serum free RPMI-1640 at 37 °C for the specified time course. In some studies, NFκB phosphorylation was examined by western (described separately) following CpG stimulation, while expression of IFN-β was examined by qPCR (described separately) in other studies. Primary DCs harvested from whole bone marrow from either *Ndst1f*/f *CD11cCre* + mutant or *Ndst1f*/f *CD11cCre*− control mice were used in separate studies examining ELISA based IFN-β protein produced in the culture medium in response to either the TLR9 ligand CpG or to the TLR7 ligand R848, described separately.

### Quantitative PCR to assess IFN-β, Ndst1, and viral RNA expression

Complementary DNA (cDNA) was generated from cell and viral samples using RNaqueaous 4-PCR Kit (Invitrogen) and Super Script III First Stand Kit (Invitrogen) based on manufacturer protocols. Quantitative PCR (qPCR) was performed with sample mixture of 100 ng of cDNA and primers for IFN-β 1 (according to manufacturer protocol; Biorad PrimePCR), and referenced to GAPDH expression. Primers were also applied for Ndst1 to determine Ndst1 silencing efficiency in some studies. While virus was not detected by plaque assay at day 5 p.i., (carried out as in^45^) we performed viral RNA qPCR to detect/quantify PR8 IAV viral genomic material from lung samples at that timepoint: For viral RNA qPCR, total RNA was isolated from mouse lung homogenates, and IAV virus (5,000,000 PFU starting material). Two separate primer sets for the H1N1 PR8 nucleoprotein^24^ and the IAV M1/M2 protein^23^ were used to quantify PR8 virus from tissue based on referencing to a standard curve generated from dilutions of the PR8 viral stock cDNA for which qPCR cycle thresholds were determined and plotted. Samples were run in the presence of iQ Sybr Green (Bio-Rad) on a StepOnePlus (Applied Biosystems) quantitative PCR machine. A quantity of 100 ng of mouse lung cDNA was added to each reaction in triplicate for quantitative analysis. Primer sequences were as follows:

Ndst1 Primers: 5′-GGACATCTGGTCTAAG-3′; reverse 5′-GATGCCTTTGTGATAG-3′

PR8 viral NP primers:

5’-GGGTGAGAATGGACGAAAAA-3’; reverse 5’-TCCATCATTGCTTTTTGTGC-3’.

PR8 viral M1/M2 primers:

5’-GGACTGCAGCGTTAGACGCT-3’; reverse 5’-CATCCTGTTGTATATGAGGCCCAT-3’.

### Flow cytometry

Whole mouse lung was digested in 0.2% collagenase solution in cell culture medium for 1 h at 37 °C. Tissue was passed though a 70 mm cell strainer at the end of digestion. Red blood cell lysis was performed on filtered material, and the cell suspension was washed in 1% BSA/PBS before antibody labeling. For studies examining for cells harboring the mouse IAV nucleoprotein (NP), samples were labeled with FITC conjugated anti-IAV NP antibody (AA5H; Abcam) at 5 μg/mL for 1 h at 4 °C in 1%BSA/PBS. Cells were washed and assayed on a CytoFlex (Beckman Coulter) flow cytometer. Analysis was performed using FlowJo software (FlowJo version 10.1). In additional studies aimed to assess inflammatory and T cell responses in the early phase of infection, we carried out analyses of AA5H NP expression in cells liberated from collagenase-digested lung samples in mice day 5 p.i.; and we found no significant difference between IAV NP viral load in mutant versus wildtype lungs at this time point (Supplemental Fig. S7). For flow cytometry examination of T cell subsets from whole lung at day 5 p.i., anti-mouse CD8a PE (Tonbo, 50–0081) was incubated at 2 μg/ml with the lung-digest cells for 1 h on ice. Unlabeled cells and isotype-match secondary antibody were used as controls; and flow cytometry was used to determine % CD8 + T cells in the lung digest cell population. To quantify CD4 + T cells, anti-mouse CD4 antibody (Invitrogen) was used at 2 μg/ml; and antibody against the FOXP3 + T cells (Invitrogen) was used at 2 μg/ml to quantify the T_reg_ subset population (as a % of CD4 + T cells).

### Protein preparation

Cells were lysed in RIPA buffer (Teknova) containing 1 mM sodium orthovanadate, 1 mM PMSF, and 10 µl/ml protease inhibitor cocktail at − 20 °C. After equilibrating at 4 °C, protein was collected by centrifugation at 12,000 rpm for 15 m. Concentrations of isolated protein samples were quantified using Micro BCA Protein Assay Kit (Thermo Fisher).

### ELISA assays

Mouse primary bone marrow DCs from *Ndst1f*/f *CD11cCre* + mutant mice and *Cre*− control mice matured in culture in the presence of GM-CSF for a total of 9 days were exposed to Resiquimod (R848) (Invivogen), which was added to the cells at 0, 0.1, 1.0, or 10 μg/mL in 12-well plates at the start of Day 8, and incubated for 24 h prior to assay on day 9, when cells were collected and spun down for measurement of IFN-β in supernatants. In some assays, cells at day 8 in culture were exposed to CpG (Invivogen; CpG class B ODN) at 1 μM concentration overnight prior to assay at day 9. For assay measurements on culture day 9, 50 μL of supernatant was removed in triplicate, and assayed for INF-β using a LumiKine mINFβ detection kit (Invivogen). The assay was performed according to manufacturer’s protocol using PBS as the coating buffer.

### Western blotting

Isolated protein samples were run on a 4–15% protein gel in Tris/Glycine/SDS buffer (BIO-RAD) at 180 V for 30 min and transferred onto 0.2 µm Nitrocellulose filter paper sandwiches (BIO-RAD) in 100 mM CAPS and 20% methanol at < 8 °C and 30 V for 2 h. Membranes were then rinsed with Tween-20/TTBS and blocked in Odyssey Blocking buffer (LI-COR) for 1 h. Primary antibody labeling was carried out with anti-phosphorylated NFκB p65 and anti- NFκB p65 (Cell Signaling 3031S and 4764S, respectively) (diluted to 1:1000; 88 ng/ml in Odyssey Blocking buffer) on a shaker overnight at 4˚C. Membranes were rinsed four times with TTBS for 15 min each, and then labeled with IRDye 800CW goat anti-rabbit IgG secondary antibody (LI-COR) (diluted to 1:10,000 in Odyssey Blocking buffer) on a shaker in the dark at RT for 1 h. Membranes were rinsed with TTBS again, and air-dried in the dark. Final blotting results were resolved using a LI-COR Odyssey system. (Full-length blot image for gel bands shown in Fig. 2B is shown in Supplemental Figure S8.)

### Statistics

For most comparisons of mean values, the unpaired student’s T-test was used, while in one analysis comparing group proportions, the chi-square statistic was used. The paired student’s T-test was used in analyses comparing means involving paired data (e.g., comparing paired data from multiple repeated experiments). A value of P < 0.05 was used to determine statistical significance.

## Supplementary Information

Supplementary Information.
