# Supplementary material for: Genetic alteration of heparan sulfate in CD11c + immune cells inhibits inflammation and facilitates pathogen clearance during influenza A virus infection
Source: Sci Rep. 2022 Mar 30;12:5382. doi: 10.1038/s41598-022-09197-7 (PMC8968721; doi:10.1038/s41598-022-09197-7)
Supplement: Supplementary file 1 — Supplementary Information. [file 41598_2022_9197_MOESM1_ESM.pdf]

Supporting Information:

Genetic alteration of heparan sulfate in CD11c+ immune cells inhibits inflammation and facilitates pathogen clearance during influenza A virus infection

So Young Kim,[1,2] Purva Gupta,[1,2] Scott C. Johns,[2,6] Elina I. Zuniga[3], John R. Teijaro[4], and Mark M. Fuster[2,1,5,6]

[1]Department of Medicine, Division of Pulmonary and Critical Care, University of California San Diego, La Jolla, CA, USA. [2]VA San Diego Healthcare System, Medical and Research Sections, La Jolla, CA, USA. [3]Division of Biological Sciences, University of California San Diego, La Jolla, CA; USA. [4]The Scripps Research Institute, La Jolla, CA, USA. [5]Glycobiology Research and Training Center, University of California San Diego, La Jolla, CA, USA. [6]Veterans Medical Research Foundation, San Diego, CA, USA

Correspondence: Mark M. Fuster, MD, VA San Diego Healthcare System and UCSD Department of Medicine, Division of Pulmonary & Critical Care, 3350 La Jolla Village Drive, San Diego, CA 92161-111J (Telephone: 858/552-8585x7347, or 858/699-8935; FAX: 858/546-1754; E-mail: mfuster@health.ucsd.edu).

Supplemental Figure S1. Lung inflammation at day 5 post- IAV inoculation in *Ndst1*f/f *CD11cCre*+ mutant and *Ndst1*f/f *CD11cCre*- wildtype mice. Mutant and wildtype mice were inoculated with an intranasal dose of 20 PFU of PR8 IAV (*n*=3 mutant and n=3 wildtype) in a cohort of mice of similar age to those examined at day 9 post-inoculation (p.i.) in main Fig.1. At day 5 p.i., lungs were harvested, inflated, paraffin embedded, and sectioned; and histopathology from the lung sections was analyzed by a trained pathologist blinded to genotype. The mean inflammatory index for each lung (estimated on a 0 – 3 scale of severity) was normalized from inflammatory scores of inflamed areas on lung sections. Inflammatory indices were assessed through analyses of both pathologic intensity of inflammation as well as the mean spatial inflammatory involvement of lung sections. Spatial inflammatory involvement for any given lung histopathologic section (percentage of section with any inflammation) was also initially determined through quantification by counting blinded to genotype. Mean +/- SD for the measures normalized to wildtype is shown on the graph. There were no significant differences between mean values at this time-point.

Supplemental Figure S2. Mean percentage weight loss relative to baseline in *Ndst1*f/f *CD11cCre*+ mutant and *Ndst1*f/f *CD11cCre*- wildtype mice intranasally administered with inoculum of 20 PFU of A/Puerto Rico/8/1934(H1N1) (PR8 IAV). Mice were observed daily for signs of illness and weighed at the indicated points during a 9-day period following experimental inoculation with IAV. Graph shows the weight trends over 9 days for the cohort of mice (n=10/genotype) for which ultimate IAV viral NP is reported at the end of the 9 day period (corresponding to day 9 p.i. NP IHC data in main Fig.3A). Statistics reported for final weight trend (Δ weight) over 9 day period referenced to baseline were: Chi-square statistic (2 x 2 for

presence/absence of >10% weight loss over study period and presence/absence of mutation; *P*=0.07), and Mann-Whitney U test for degree of weight loss >10% in wildtype (5.6%) versus mutant (2.9%) mice at day 9 (**P*=0.001 for difference).

Supplemental Figure S3. Ndst1 silencing efficiency in DC2.4 cells measured by qPCR. Percent Ndst1 expression was normalized to GAPDH expression, with measures run in triplicate. Mean expression +/- SD is plotted for si(Ndst1) cells versus that for cells treated with scrambled-vehicle si(Control) RNA; **P*=0.038 for the difference in means.

Supplemental Figure S4. Assessment of NFkB activation by the TLR7 agonist R848 in primary *Ndst1*f/f *CD11cCre*+ mutant and *Cre*- wildtype bone marrow derived DCs in culture. Cells were stimulated with R848 (1 μg/ml) for the indicated times. The level of phosphorylated NFκB subunit p65, as measured by western blotting of cell lysates, was indexed to the corresponding total p65 value, and the graphed indexed values for each condition (at baseline and at 15 min and 1hr post stimulation) are shown normalized to that of un-stimulated cells in the graph. Data is shown for n=3 independent experiments. No significant differences between mean mutant and wildtype responses were noted at baseline or at subsequent time points for stimulation.

Supplemental Figure S5. Photomicrographs of IHC stained lung sections using antibody against the IAV AA5H Nucleoprotein to detect viral NP density in lung tissue: Panel (A) shows lung sections from a uninfected *Ndst1*f/f *CD11cCre*- wildtype control mouse treated without primary antibody (left), and that from a control mouse stained with primary antibody (AA5H NP) and all secondary reagents (right). (B) Representative IHC-stained lung section from

IAV-infected wildtype mouse (at day 9 p.i.) stained with primary anti-AA5H NP antibody. (C) Representative lung section from IAV-infected *Ndst1*f/f *CD11cCre+* mutant mouse stained with primary anti-AA5H NP antibody. Magnification, X 400; scale bar = 20 μm.

Supplemental Figure S6. Total CD4+ T cells from whole-cellular mouse lung digests from *Ndst1*f/f *CD11cCre+* mutant and *Ndst1*f/f *CD11cCre-* control mice at day 5 p.i. were quantified by flow cytometry: Whole cellular digests from lungs of mice sacrificed at day 5 p.i. were examined by flow cytometry using antibody against the CD4+ T cells, and measured on a CytoFlex (Beckman Coulter) flow cytometer. Graph shows data from n=5 mutant and n=4 wildtype mice, with no significant differences in means.

Supplemental Figure S7. IAV nucleoprotein in whole-cellular mouse lung digests was quantified by flow cytometry: Whole cellular digests from lungs of mice sacrificed at day 5 p.i. were examined by flow cytometry using antibody against the PR8 IAV AA5H nucleoprotein (NP) antibody, measured on a CytoFlex (Beckman Coulter) flow cytometer. (A) A typical histogram is shown, wherein control (non-labeled cells) are represented by light grey curve to the left, with isotype-matched antibody control shown as solid-grey curve in center; and dark curve indicating +AA5H stained cells, showing AA5H-positive tail to the far right (bar for which % positive whole-lung digested cells ranged from 0.8% to 3%). (B) Analysis of mean AA5H NP expression, showing % positive cells, for n=3 mice per genotype in this experiment assessed at day 5 p.i., and showed no significant difference between IAV NP viral load in mutant versus wildtype lungs at this time point (plotted on bar-histogram). Comparing this data to that of IHC quantified data from mice examined at day 5 p.i. (Fig.3A, graph to left) shows similar results.

Supplemental Figure S8. Full-length western blot lanes are presented for representative blot shown in main Figure 2B. The blots show full-length gel images demonstrating immuno-blot bands for phospho-p65 (upper blot) and total p65 (lower blot) collected from DC2.4 cells at baseline (-) or stimulated with 1μM CpG for 15 min and 1h; and stimulation in the absence or presence of Ndst1 silencing denoted by "–" or "+" above each lane.

Supplemental Table 1: Summary of Histologic Inflammatory Scoring in Figure 1 and Supplemental Figure S1.

Day 9 post-inoculation data: Corresponding to Figure 1C

**Inflammatory Intensity***	Mean Value	(St.Dev.)	**Spatial Inflammation#**	Mean Value	(St.Dev.)
*Ndst1*f/f *CD11cCre*- (WT)	2.5	(0.5)	*Ndst1*f/f *CD11cCre*- (WT)	35%	(17%)
*Ndst1*f/f *CD11cCre*+ (Mut)	1.4	(0.8)	*Ndst1*f/f *CD11cCre*+ (Mut)	11%	(5.7%)
P-value difference	0.03		P-value difference	0.02	
Normalized Data (Graphed in Figure 1)	WT 1.0<br>Mut 0.56	(0.2)<br>(0.32)	Normalized Data (Graphed in Figure 1)	WT 1.0<br>Mut 0.31	(0.48)<br>(0.17)

Day 5 post-inoculation data: Corresponding to Supplemental Figure S1**

**Inflammatory Intensity***	Mean Value	(St.Dev.)	**Spatial Inflammation****	Mean Value	(St.Dev.)
*Ndst1*f/f *CD11cCre*- (WT)	2.0	(0.8)	*Ndst1*f/f *CD11cCre*- (WT)	37%	(9%)
*Ndst1*f/f *CD11cCre*+ (Mut)	2.7	(0.5)	*Ndst1*f/f *CD11cCre*+ (Mut)	55%	(15%)
P-value difference	0.21		P-value difference	0.37	
Normalized Data (Graphed in Supp FigS1)	WT 1.0<br>Mut 1.33	(0.4)<br>(0.24)	Normalized Data (Graphed in Supp Fig.S1)	WT 1.0<br>Mut 1.49	(0.24)<br>(0.4)

* Criteria for measurement: Possible range from 0 (min) to 3 (max) on relative intensity scale used for estimating degree of inflammation on H&E stained slide by Pathologist blinded to genotype (whole-lung slide for each mouse).

** Criteria for measurement: Partial extent of inflammation (% area covered) on lung histologic section determined by Pathologist blinded to genotype (whole-lung slide for each mouse).

** Quantification of AA5H nucleoprotein and T cells in IHC tissue sections was carried out by counting of 5 high power fields (HPF) per histologic section through whole lung by two independent technical associates blinded to slide information.

Supplemental Table 2: Detection of IAV M1/M2 viral-protein RNA by quantitative PCR using whole-lung digest samples from wildtype and mutant mouse lungs at day 5 post-inoculation with PR8 IAV. Cycle thresholds (Ct) are shown at right for detection of viral RNA by qPCR in extractions from whole lung homogenates, with Ct of 30.6 corresponding to lung from a non-infected mouse. A standard viral dilution from a PR8 IAV stock in smaller sub-table at left shows Ct values corresponding to dilutions from an arbitrary 1x stock concentration of PR8 virus (and a no-virus control at bottom).

**PR8 viral stock aliquot***	**qPCR cycle threshold (M1/M2 IAV RNA detection)**
1x stock	24.0
10-fold dilution	27.2
100-fold dilution	30.4
No virus control	36.0

**Mouse genotype and lung sample preparation****	**qPCR cycle threshold (M1/M2 IAV RNA detection)**
Control (no virus) mouse	30.6
*Ndst1*f/f *CD11cCre*- WT #1	28.2
WT #2	25.2
WT #3	23.8
WT #4	18.9
*Ndst1*f/f *CD11cCre*+ Mut #1	(>30.6)
Mut #2	(>30.6)
Mut #3	(>30.6)
Mut #4	22.2
Mut #5	19.1
Mut #6	18.7

* Arbitrary PR8 viral stock with viral concentration 500,000 PFU per mL.

** Whole lung cDNA used in QPCR analysis was obtained from purified lung RNA (extracted from a conserved lung lobe for each mouse), with 100ng of cDNA used in for each qPCR reaction.

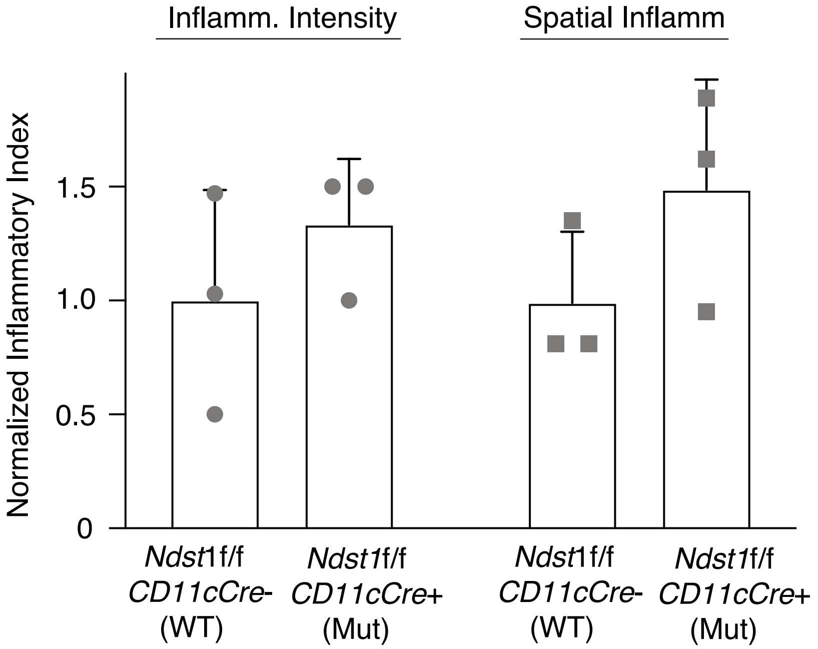
Inflamm. Intensity
Spatial Inflamm
Normalized Inflammatory Index
0
0.5
1.0
1.5
Ndst1f/f
CD11cCre-
(WT)
Ndst1f/f
CD11cCre+
(Mut)
Ndst1f/f
CD11cCre-
(WT)
Ndst1f/f
CD11cCre+
(Mut)

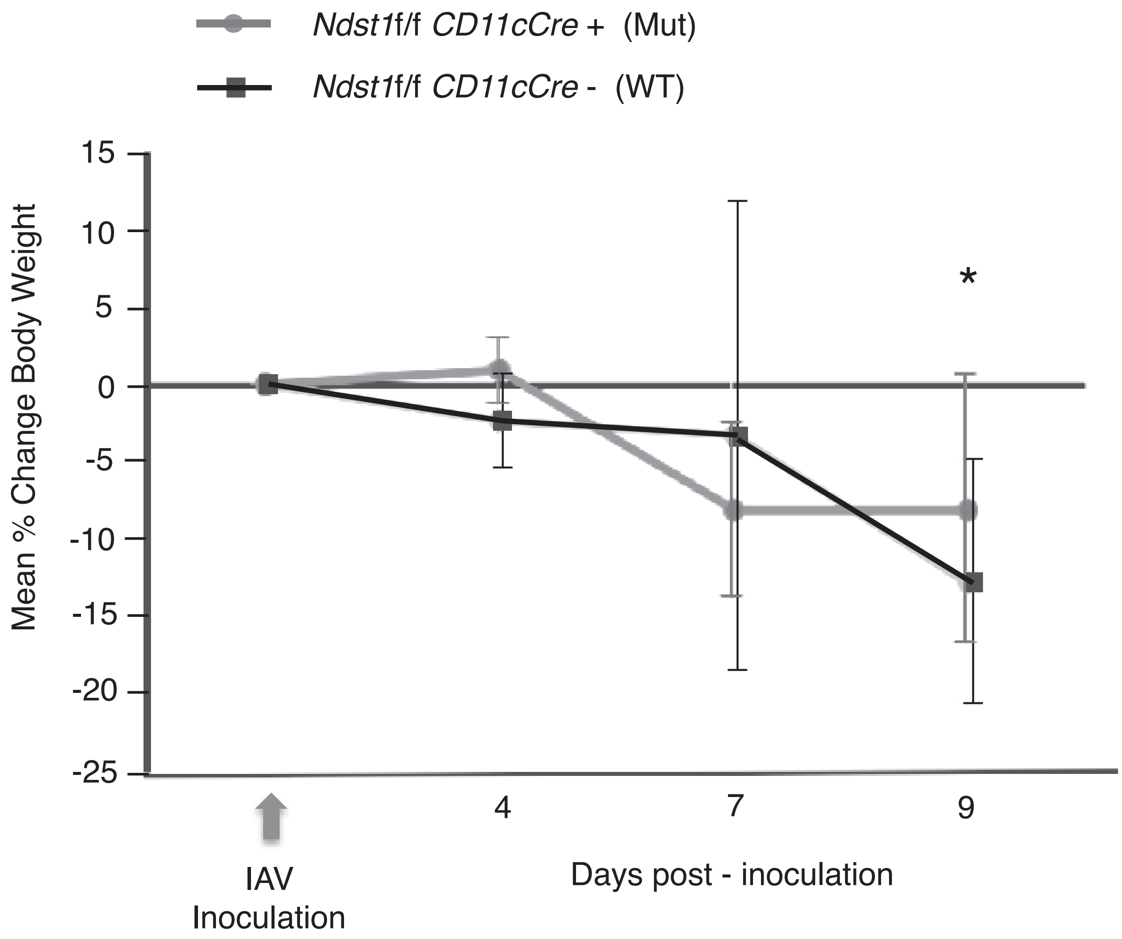
Ndst1f/f CD11cCre + (Mut)
Ndst1f/f CD11cCre - (WT)
Mean % Change Body Weight
15
10
5
0
-5
-10
-15
-20
-25
*
4
7
9
IAV Inoculation
Days post - inoculation

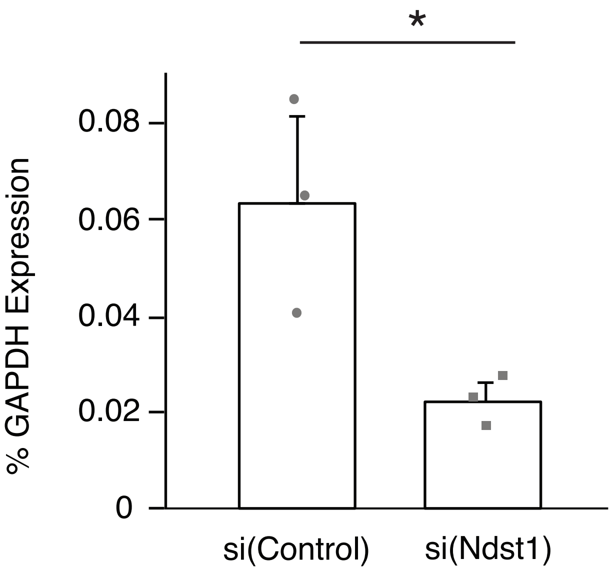
*
% GAPDH Expression
0.08
0.06
0.04
0.02
0
si(Control)
si(Ndst1)

Phospho-p65 (NF-κB activation) CpG responses

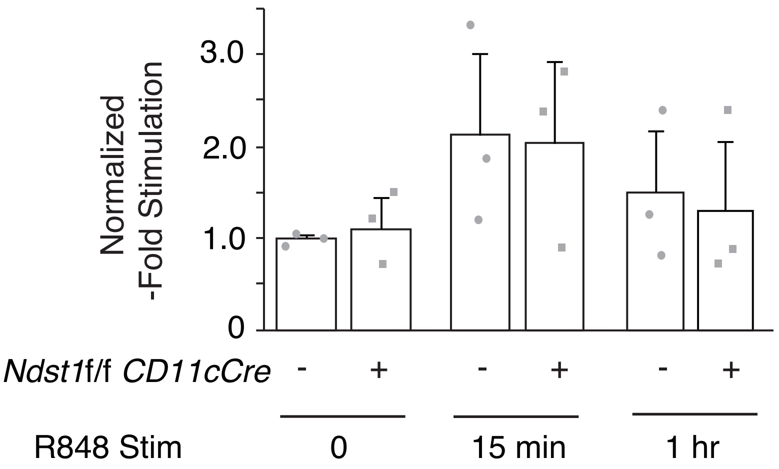

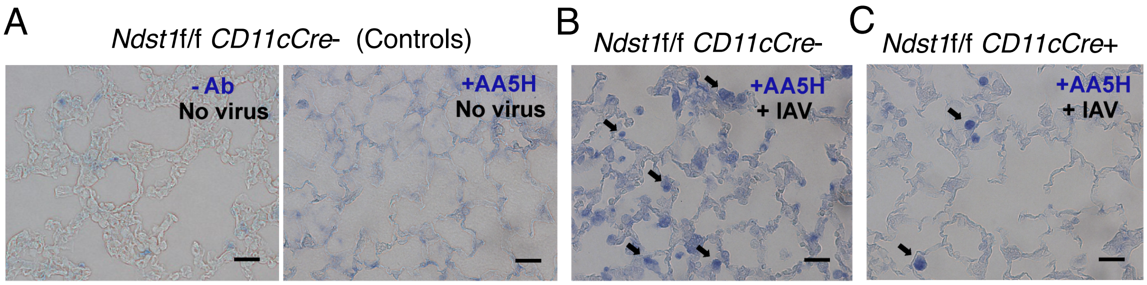
A
*Ndst1*f/f *CD11cCre*- (Controls)
- Ab
No virus
+AA5H
No virus
B
*Ndst1*f/f *CD11cCre*-
+AA5H
+ IAV
C
*Ndst1*f/f *CD11cCre*+
+AA5H
+ IAV

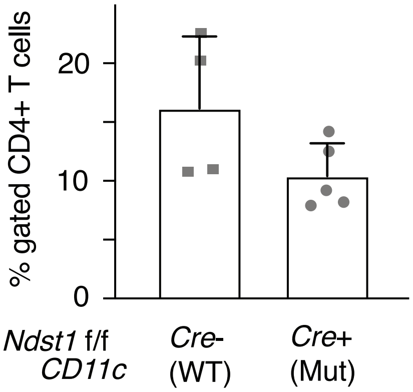
% gated CD4+ T cells
20
10
0
Ndst1 f/f
CD11c
Cre-
(WT)
Cre+
(Mut)

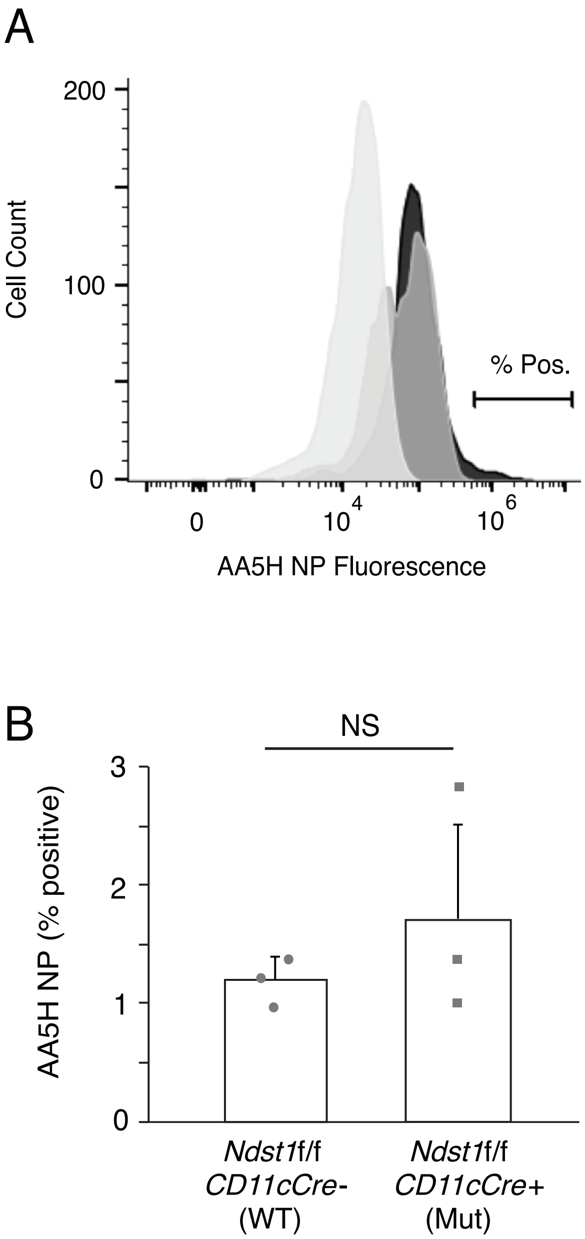
A
200
100
0
Cell Count
% Pos.
0
10^4
10^6
AA5H NP Fluorescence
B
NS
3
2
1
0
AA5H NP (% positive)
Ndst1f/f
CD11cCre-
(WT)
Ndst1f/f
CD11cCre+
(Mut)

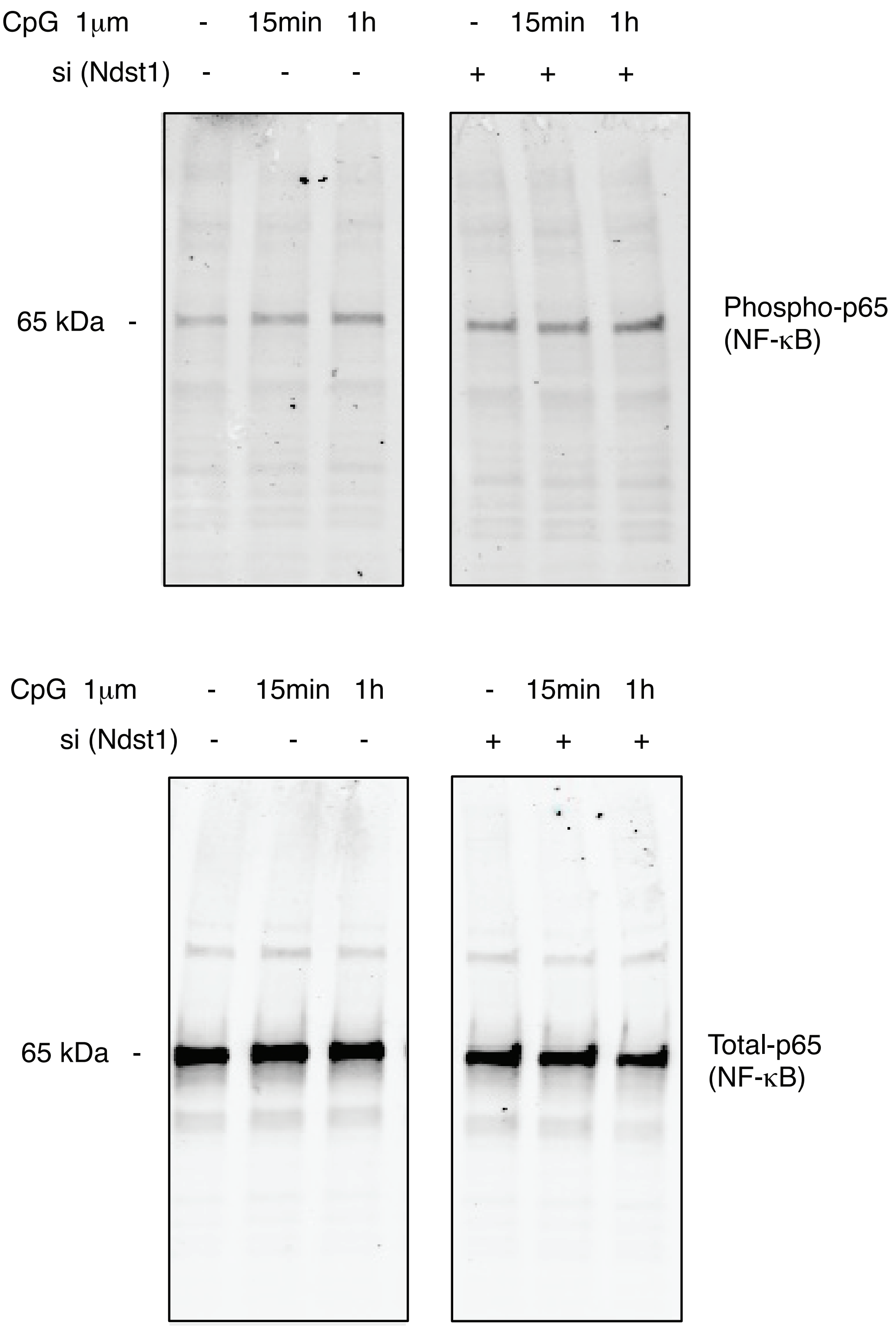
CpG 1μm - 15min 1h - 15min 1h
si (Ndst1) - - - + + +
65 kDa -
Phospho-p65
(NF-κB)
CpG 1μm - 15min 1h - 15min 1h
si (Ndst1) - - - + + +
65 kDa -
Total-p65
(NF-κB)
